# INNOVATIVE TECHNOLOGIES: Microscopy Not to Dye For

**DOI:** 10.1289/ehp.118-a21

**Published:** 2010-01

**Authors:** Tim Lougheed

**Affiliations:** **Tim Lougheed** has worked as a freelance writer in Ottawa, Canada, since 1991. A past president of the Canadian Science Writers’ Association, he covers a broad range of topics in science, technology, medicine, and education

Users of confocal microscopes now have access to a relatively inexpensive module that can be readily installed to dramatically increase the capabilities of these instruments. Members of Canada’s National Research Council (NRC) initiated the commercial development of this technology, hoping to provide researchers with cost-effective access to imaging strategies with distinct advantages for some applications.

The price tag for the new system will be about US$57,000, a fraction of what it takes to acquire such a microscope. This new investment makes it possible to create detailed images of fatty structures that are typically nonfluorescent, such as the fibrous caps responsible for atherosclerosis and the myelin sheath surrounding nerve structures. Importantly, the method is label-free, meaning it does not rely on any dyes or stains. Moreover, images can be created in seconds rather than hours, offering real-time *in-vivo* representation of such complex processes as a virus infecting cells.

The technology is called coherent anti-Stokes Raman scattering, or CARS. Developed in the mid-1960s as a spectroscopic tool by materials engineers at the Ford Motor Company, in the late 1990s the concept was extended to include microscopy. The technique builds on the same hardware used in confocal imaging, consisting of two lasers plus an elaborate array of lenses and mirrors. When this bright light reaches a specimen, it fluoresces to a significant depth, and with the proper resolution, yields a detailed three-dimensional image.

Taking advantage of these same two lasers, CARS adds another light field based on a difference in frequency between these light sources, a difference that precisely matches a target molecule’s vibrational resonance. The resulting image can be obtained even as the rest of the microscope generates a standard confocal image.

In 2007 NRC researcher Albert Stolow and his colleagues demonstrated that CARS images could be obtained using the same laser technology already found in confocal microscopes. The work taking place at the NRC also caught the attention of optics giant Olympus, which saw the prospect of a new product that would build on an established market of microscope users without asking them to convert exclusively to unfamiliar methodology or acquire another expensive, specialized piece of equipment. The company subsequently partnered with Stolow’s team to come up with the design for an add-on device that would mate seamlessly with the company’s existing confocal systems.

The enhanced 300-nm spatial resolution of CARS is achieved without dyes or stains to provide the necessary contrast. This feature makes it easier to work with living tissue that might diffuse or react with these agents and compromise the outcome.

That is a big selling point for Michael Sowa, an NRC researcher specializing in biodiagnostic spectroscopy and heart disease. CARS allows him to visualize fibrous patches of plaque building up inside veins and arteries; specifically, he can look for lesions that could burst and release material that could cause a stroke. “It’s a really powerful tool for investigating vascular walls, and atherosclerosis in particular,” he says.

NRC investigator John Pezacki was one of the first to adopt CARS almost a decade ago. Before then, he would have relied primarily on fixed cell staining, which amounted to taking a handful of snapshots portraying a complex chemical interaction while whatever might be happening in between each picture remained unknown. “It’s the equivalent of doing very good quality photography versus cinematography,” he explains. “It allows us to do kinetic measurements, answering the question of when [events] happen during the viral life cycle.”

And while the CARS system currently performs optimally with the molecules making up lipids, Pezacki envisions potential for this technology in environmental analysis. “The holy grail of this area going forward is to be able to do lower-abundance materials, such as peptides, proteins, and nucleic acids,” he says. “If you can . . . look at the unique CARS signals from [natural products or drugs] and get a label-free contrast image of where those things are in the cell or in a tissue, then you have the opportunity to do that with environmental toxicants as well.”

